# Microbial tryptophan metabolites modulate blood-brain and gut barriers *in vitro*

**DOI:** 10.1016/j.nsa.2025.106876

**Published:** 2025-11-21

**Authors:** Cristina Rosell-Cardona, Emily G. Knox, Paula Sánchez-Díaz, Sarah-Jane Leigh, Emanuela Tirelli, Michael S. Goodson, Nancy Kelley-Loughnane, María R. Aburto, Sarah Kittel-Schneider, John F. Cryan, Gerard Clarke

**Affiliations:** a APC Microbiome Ireland, University College Cork, Cork, Ireland; b Department of Anatomy and Neuroscience, University College Cork, Cork, Ireland; c Department of Psychiatry and Neurobehavioural Science, University College Cork, Cork, Ireland; d 711th Human Performance Wing, Air Force Research Laboratory, Wright-Patterson Air Force Base, Dayton, OH, USA; e Department of Psychiatry, Psychotherapy and Psychosomatic Medicine, University Hospital Würzburg, Würzburg, Germany

## Abstract

The gut microbiota influences brain function via the gut-brain axis, but the underlying molecular processes remain unclear. Critical to this communication are barrier systems, such as the epithelial gut and the blood-brain barrier, which mediate the interface between circulating signals and gut-brain communication. Microbial metabolites are key mediators of the gut microbiota that can influence barrier integrity. In this study, we used well-established *in vitro* models of the blood-brain and gut barriers and exposed them to a wide range of physiologically relevant stress-associated microbial metabolites, including tryptophan-derived metabolites with and without lipopolysaccharide (LPS) as a disrupting insult. We demonstrated that indole, indole-3-acetate, indole-3-propionate and tryptamine can modulate both gut and brain barriers in a dose- and cell-type dependent manner. Our findings suggest that specific indole metabolites should be further evaluated as promising novel therapeutic interventions to regulate barrier integrity along the microbiota-gut-brain axis.

## 1 Introduction

The microbiota-gut-brain axis facilitates bidirectional communication between the gut microbiota and the central nervous system, influencing host physiology, behavior, and brain function (Cryan et al., 2019). Among the key mediators of this communication are microbial metabolites, diverse bioactive molecules produced by the gut microbiota (Ahmed et al., 2022). These metabolites are increasingly recognized not only for their systemic effects but also for their direct impact on critical barriers of the microbiota-gut-brain axis, including the intestinal epithelial barrier and the blood-brain barrier (BBB) (Parker et al., 2020). These barriers act as dynamic gatekeepers, regulating the passage of microbial and molecular signals into systemic circulation and the central nervous system (Aburto and Cryan, 2024).

While many studies have investigated how microbial metabolites affect gut barrier function (Ghosh et al., 2021) much less is known about their direct effects on the BBB. In germ-free mouse models, the absence of microbiota has been shown to increase BBB permeability and reduce the expression of tight junction proteins, effects that persist from prenatal stages into adulthood (Braniste et al., 2014). Moreover, barrier disruption in both the gut and brain has been implicated in a range of pathological conditions, suggesting that impaired barrier function may represent a shared mechanism underlying gut-brain comorbidities (Sweeney et al., 2019; Verma et al., 2013).

There are several external and internal factors that can lead to an impaired barrier function, like inadequate nutritional support and environmental and psychosocial stress (Person and Keefer, 2021; Segarra et al., 2021). Stress is a well-known modulator of the microbiota-gut-brain axis (Cryan and Dinan, 2012; Sudo et al., 2004). Chronic stress has been linked to altered gut microbiota composition in humans (Almand et al., 2022) and animal models (Cruz-Pereira et al., 2022; Foster et al., 2017), along with disrupted gut motility and increased intestinal permeability (Kelly et al., 2015). Similarly, prolonged stress exposure impairs BBB integrity (Welcome and Mastorakis, 2020). Several studies have shown that stress-induced impairment of blood-brain barrier (BBB) integrity is accompanied by increased peripheral and neuroinflammation, which may contribute to barrier disruption, a key hallmark in the development of mental and neurodegenerative disorders (Welcome and Mastorakis, 2020; Carvey et al., 2009). There is now increasing research showing an upregulation of the immune response by acute stress, potentially marking a transitional phase that bridges acute and chronic stress, during which sustained immune activation may contribute to maladaptive changes over time (Rohleder, 2019). However, the effects of acute stress on the interplay between microbial metabolites and barrier function remain poorly understood. While microbial metabolites are increasingly recognized as modulators of host physiology (Ahmed et al., 2022; O'Riordan et al., 2022), research into their direct impact on barrier integrity, particularly at the BBB, remains limited (Knox et al., 2022a; Rosell-Cardona et al., 2025a; Tofani et al., 2025).

Previously published data from our lab suggest that acute stress modulates both gut permeability and tryptophan-derived metabolite levels (Gheorghe et al., 2024). These metabolites are of particular interest due to their important role in gut-brain axis signaling and their modulation by nutrition and stress (Gheorghe et al., 2019). The microbial metabolic pathways processing tryptophan in the gut were notably altered under acute stress conditions and may play a key role in regulating gut barrier function or at sites distal to their production. Therefore, tryptophan-derived indole derivatives were selected for further investigation. Using *in vitro* models of intestinal and brain barriers, we aimed to explore the potential for these metabolites to modulate barrier integrity under basal conditions and in response to a pro-inflammatory lipopolysaccharide (LPS) insult.

## 2 Material and methods

### 2.1 Cell lines

Murine brain endothelial cells (bEnd.3; CRL-2299) and human carcinoma colonic cells (T84; CCL-248) were purchased from ATCC (American Type Culture Collection, Middlesex, UK). Both cell lines were cultured in Dulbecco's Modified Eagle Medium: Nutrient Mixture F-12 (containing 10 % heat-inactivated fetal bovine serum (Sigma-Aldrich, F9665) and 1 % penicillin/streptomycin). Cells were grown on 24-well plate polyethylene terephthalate (PET) transwell inserts (surface area 0.33 cm^2^, pore size 0.4 μm; Grenier Bio-one #662641) and maintained at 37 °C and 5 % CO_2_. BEnd.3 cells (under passage 20) were seeded at 4.5∗10^3^ cells/well, while T84 cells (under passage 15) were seeded at 1∗10^5^ cells/well. First, confluent cells were treated in the apical compartment of the transwell with microbial metabolites diluted in complete media at a range of concentrations (bEnd.3: 0, 0.1, 0.5, 1, 10, 100, 1000 μM; T84: 0, 1, 10, 100, 1000, 2500 μM). After 24hrs of metabolite pre-treatment, a pro-inflammatory LPS *Escherichia coli* O26:B6 insult was applied for an additional 24 h to disrupt the barrier integrity. This model allows us to assess the potential disruptive, protective or restorative effects of indole metabolites under basal conditions (without LPS) or pro-inflammatory conditions (with LPS). LPS concentration was optimized to be sufficient to induce a disruption (1 μg/mL for bEnd.3 cells; 300-500 μg/mL for T84 cells, yielding a significant reduction in transepithelial/transendothelial electrical resistance (TEER) of at least of 20 %, Sigma-Aldrich, #L8274), without significantly compromising cell viability **(**threshold of 80 %, Supplementary Fig. 1) (Rosell-Cardona et al., 2025a). Control wells and LPS control wells were treated with 0 μM metabolite dissolved directly in Milli-Q water, ethanol (EtOH), or DMSO (D8418-250ML) before further dilution into complete media.

### 2.2 Chemicals

To identify microbial metabolites for analysis (Table 1), we curated a list from a previous *in vivo* experiment assessing the impact of acute restraint stress on the cecal and colonic mucosal metabolome (Gheorghe et al., 2024) and also included metabolites identified by literature review based on biological plausibility to have a possible effect on barriers integrity (https://hmdb.ca/).

**Table 1:** Metabolites information.

Metabolite	Reconstitution solvent	Manufacturer	Product code
Tryptamine	EtOH	Sigma-Aldrich	#193747
Indole	DMSO	Sigma-Aldrich	#I3408-25G
Oxindole (2-oxindole)	EtOH	Sigma-Aldrich	#9808
Indole-3-propionate	EtOH	Sigma-Aldrich	#57400-5G-F
Indole-3-acetate	EtOH	Sigma-Aldrich	#S8625
Indole-3-lactate	Milli-Q water	Sigma-Aldrich	#I5508
Isatin	DMSO	Sigma-Aldrich	#114618
Skatole	DMSO	Sigma-Aldrich	#W301912

### 2.3 Trans-epithelial/Endothelial Electrical Resistance (TEER)

Transepithelial/transendothelial electrical resistance (TEER) measurements indicate transcellular and paracellular permeability to ions using a world Precision Instrument Epithelial volt/Ohm Meter 2 and chopstick electrode (Srinivasan et al., 2015). TEER is a technique used to measure the integrity of tight junction dynamics in endothelial and epithelial cell monolayers and was used as an index of paracellular and transcellular permeability, following (Rosell-Cardona et al., 2025a).

### 2.4 Permeability FITC assay

Paracellular permeability was determined following a previous publication (Rosell-Cardona et al., 2025a), by adding 4 kDa Fluorescein isothiocyanate (FITC) dextran 800 μg/mL (Sigma-Aldrich #FD4) and measuring it after a 60 min incubation at 37 °C to the apical compartment of the transwell and quantifying the amount in the basolateral compartment.

### 2.5 MTT cell viability assay

Viability assay was performed following a previous publication (Rosell-Cardona et al., 2025a). Briefly, 50 μL of 0.5 mg/mL MTT (Sigma Merk #M5655-500MG) was added to each well. The plate was covered and placed in the incubator at 37 °C for 2 h to allow crystals to form. The MTT was then removed from the wells and 100 μL of DMSO was added to each well and read at absorbance 570 nm.

### 2.6 Quantification and statistical analysis

Experimental data was expressed as mean ± SEM, n = biological replicates. At least two independent experiments were performed with at least three biological replicates in each experiment. Grubb's test was used to remove any outliers from data sets. Data was analyzed by two-way analysis of variance ANOVA for treatment (Untreated, and metabolites concentrations or type of metabolites) and LPS insult followed by Dunnet's adjusted posthoc comparison to the untreated groups. A p-value of less than 0.05 was considered significant. Data analysis and visualisation were carried out using R studio and GraphPad Prism.

## 3 Results

### 3.1 Tryptophan metabolites protect brain barrier integrity in a dose-dependent manner

**Fig. 1:**
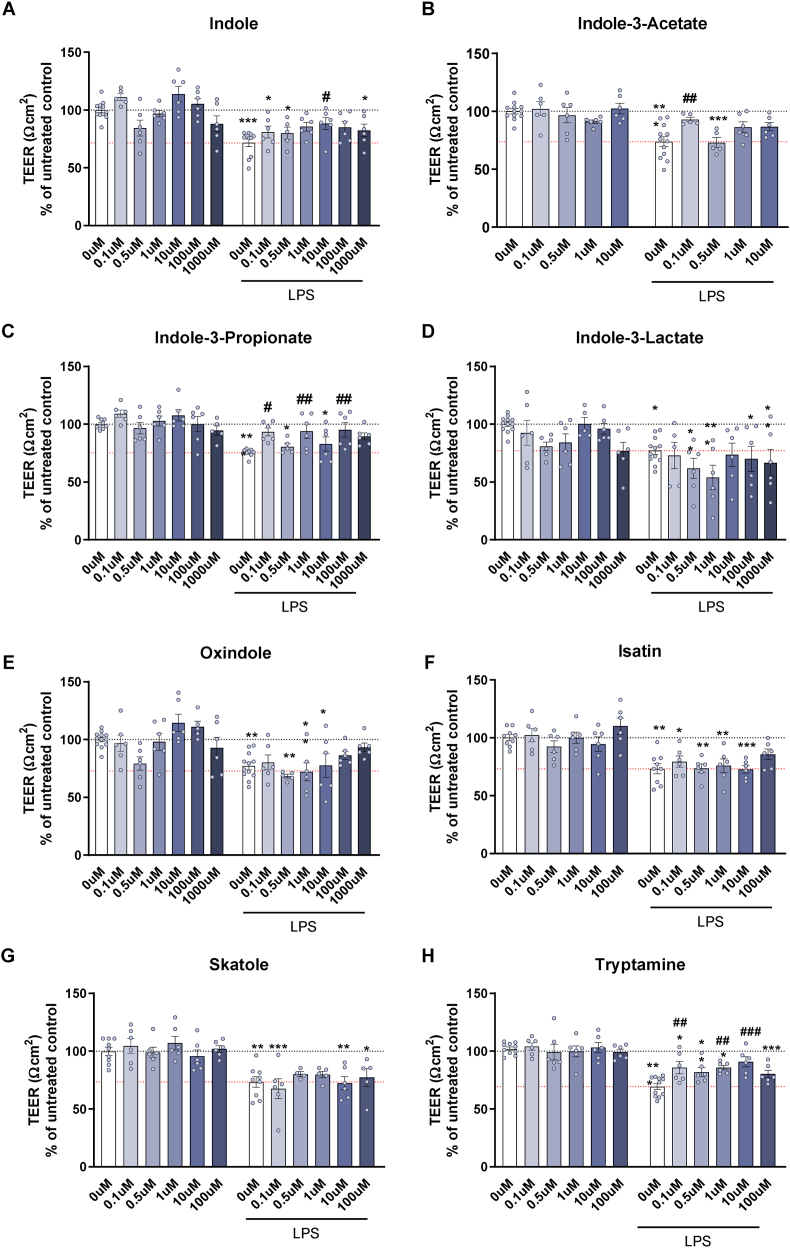
**Transendothelial electrical resistance of bEnd.3 cells following exposure to tryptophan metabolites with and without a 24 h exposure of LPS.** (A) Indole, (B) indole-3-acetate, (C) indole-3-propionate, (D) indole-3-lactate, (E) oxindole, (F) isatin, (G) skatole, (H) tryptamine. Data are mean ± SEM; two-way ANOVA followed by Dunnet's post hoc compared to untreated groups. ∗p < 0.05, ∗∗p < 0.01, ∗∗∗p < 0.001 metabolite compared to 0 μM control, #p < 0.05, ##p < 0.01, ###p < 0.001 compared to 0 μM LPS.

To understand if these metabolites have any potential role in affecting brain endothelial barrier cells, these cells were treated with the tryptophan metabolites with and without LPS. LPS significantly reduced TEER, indicating impaired barrier integrity in all experiments [all LPS, p < 0.001] (Fig. 1)*.*

Treatment with indole, indole-3-acetate, indole-3-propionate, and isatin, tryptamine improved the barrier integrity [indole, F (6,79) = 3.761, p = 0.002; indole-3-acetate, F (4,62) = 2.665, p = 0.040; indole-3-propionate, F (6,73) = 2.743, p = 0.018; isatin, F (5,66) = 2.404, p = 0.046; tryptamine, F (5,69) = 3.169, p = 0.012], and a detrimental effect was observed with oxindole and indole-3-lactate [oxindole, F (6,80) = 3.086, p = 0.009; indole-3-lactate, F (6,80) = 2.55, p = 0.025]. In the indole and tryptamine experiments, there was a significant interaction effect between LPS and the metabolite on TEER [indole and LPS, F (5,69) = 2.625, p = 0.022; tryptamine and LPS, F (5,69) = 2.557, p = 0.035]. Of these metabolites, there was a significant protection from the LPS mediated decrease in TEER with specific concentrations of indole (10 μM), indole-3-acetate (0.1 μM), indole-3-propionate (0.1, 1, 100 μM) and tryptamine (0.1, 1, 10 μM). A trend to reduce barrier function was observed with oxindole (0.5 μM), and indole-3-lactate (1000 μM without LPS; 1 μM with LPS).

**Fig. 2:**
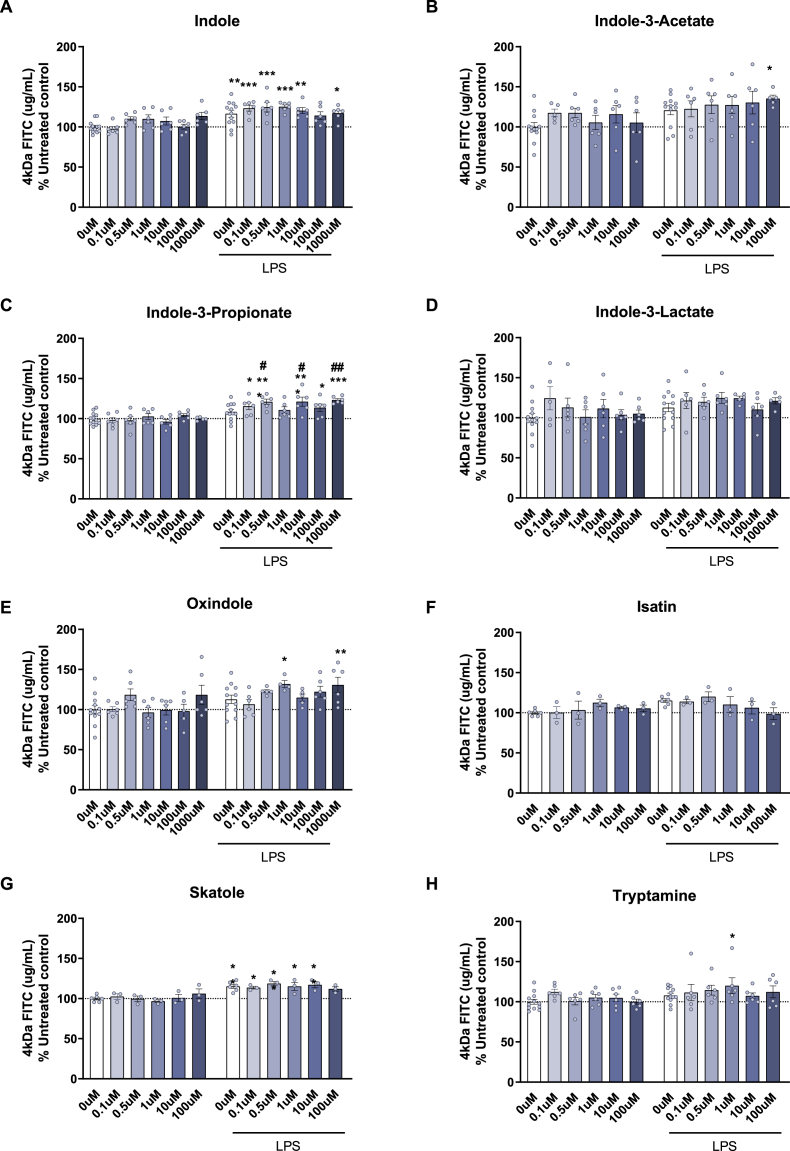
**FITC 4 kDa Permeability of bEnd.3 cells following exposure to tryptophan metabolites with and without a 24 h exposure of LPS.** (A) Indole, (B) indole-3-acetate, (C) indole-3-propionate, (D) indole-3-lactate, (E) oxindole, (F) isatin, (G) skatole, (H) tryptamine. Data are mean ± SEM; two-way ANOVA followed by Dunnet's post hoc compared to untreated groups. ∗p < 0.05, ∗∗p < 0.01, ∗∗∗p < 0.001 metabolite compared to 0 μM control, #p < 0.05, ##p < 0.01, ###p < 0.001 compared to 0 μM LPS.

Regarding FITC permeability, LPS exposure disrupted barrier integrity, leading to increased FITC-dextran permeability [LPS, p < 0.05] (Fig. 2). Treatment with indole, and oxindole showed a significant protective effect [indole, F (6,82) = 2.339, p = 0.039; oxindole, F (6,77) = 2.539 p = 0.027]. Moreover, indole-3-propionate had a detrimental effect of FITC permeability in LPS-treated cells [interaction between indole-3-propionate and LPS, F (6,81) = 2.427, p = 0.033].

Of interest, none of the concentrations used in the permeability assays reduced significantly the cell viability of bEnd.3 cells (Supplementary Fig. 2). In summary, pretreatment with indole, indole-3-acetate, indole-3-propionate and tryptamine modulated LPS-disrupting effects in a dose- and assay-dependent manner.

### 3.2 Tryptophan derived metabolites altered the gut barrier integrity in a dose-dependent manner

To explore the effects of indole metabolites on gut barrier integrity, we measured TEER, permeability to 4 kDa FITC-dextran permeability and the cell viability in T84 cells after a 48 h exposure to different indole concentrations with or without a 24 h LPS exposure.

**Fig. 3:**
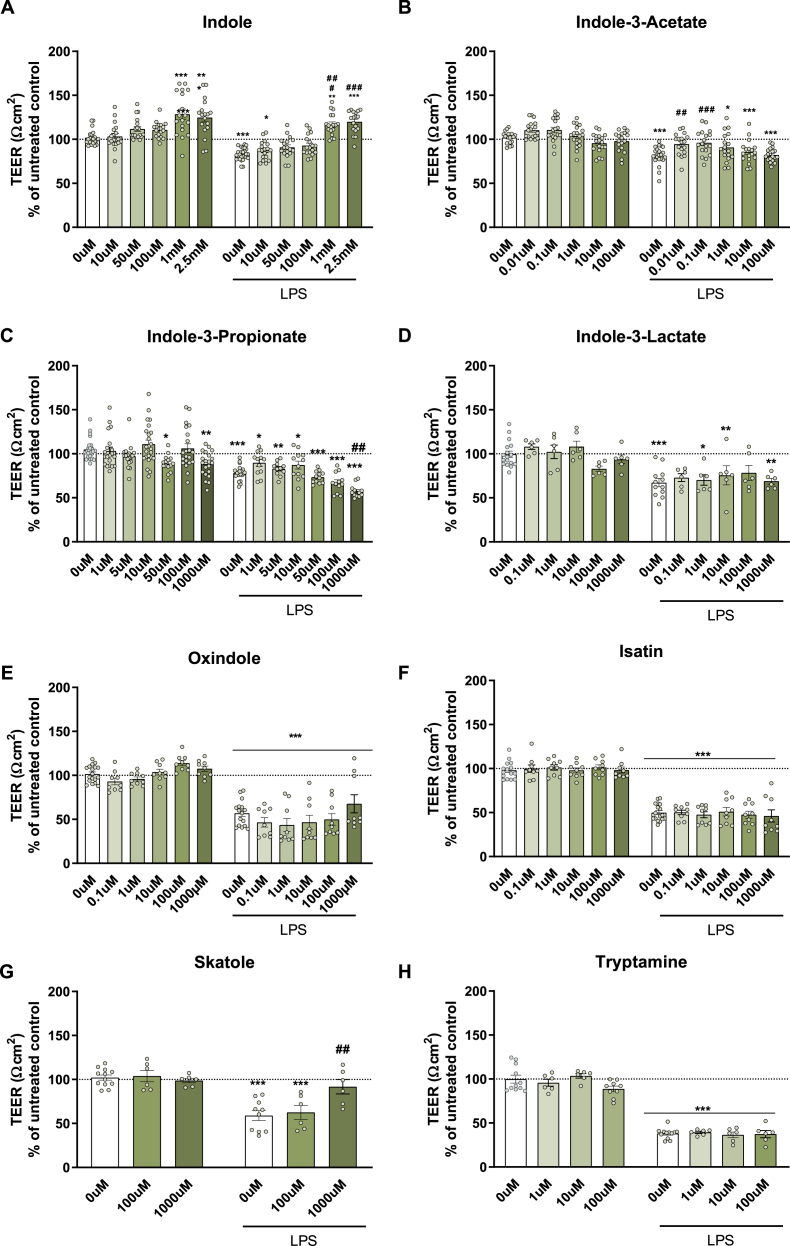
**Transepithelial electrical resistance of T84 cells following exposure to tryptophan metabolites with and without a 24 h exposure of LPS.** (A) Indole, (B) indole-3-acetate, (C) indole-3-propionate, (D) indole-3-lactate, (E) oxindole, (F) isatin, (G) skatole, (H) tryptamine. Data are mean ± SEM; two-way ANOVA followed by Dunnet's post hoc compared to untreated groups. ∗p < 0.05, ∗∗p < 0.01, ∗∗∗p < 0.001 metabolite compared to 0 μM control, #p < 0.05, ##p < 0.01, ###p < 0.001 compared to 0 μM LPS.

In all experiments, LPS insult disrupted barrier integrity resulting in a reduction of TEER: [in all LPS, p < 0.001] (Fig. 3). Treatment with indole, indole-3-acetate, oxindole and skatole prevented the LPS-induced barrier disruption in TEER [indole, F (5,215) = 37.920, p < 0.001; indole-3-acetate, F (5,208) = 10.0, p < 0.001; oxindole, F (5,112) = 3.62, p = 0.004; skatole, F (1,40) = 54.117, p < 0.001], however, indole-3-propionate had a detrimental effect of (F (6,211) = 11.365, p < 0.001). Moreover, there was a significant interaction between LPS and indole-3-propionate, and LPS and skatole [indole-3-propionate∗LPS, F (6,221) = 2.988, p = 0.008; skatole∗LPS, F (2,40) = 5.980, p = 0.005]. Of these metabolites, post-hoc analysis indicated that indole at 1 mM, and 2.5 mM increased TEER compared to the untreated control. Moreover, there was also a protective effect against LPS-disrupting effects on gut integrity in a dose-dependent manner with indole (1 mM, and 2.5 mM), indole-3-acetate (0.01 μM), and skatole (0.1, and 1000 μM). Conversely, indole-3-propionate at 1000 μM reduced the TEER on its own compared to the untreated group and in LPS-treated cells compared to LPS control. In summary, pretreatment with indole, indole-3-acetate, and skatole significantly prevented LPS-induced gut barrier disruption, whereas high-dose indole-3-propionate worsened LPS-induced barrier impairment.

**Fig. 4:**
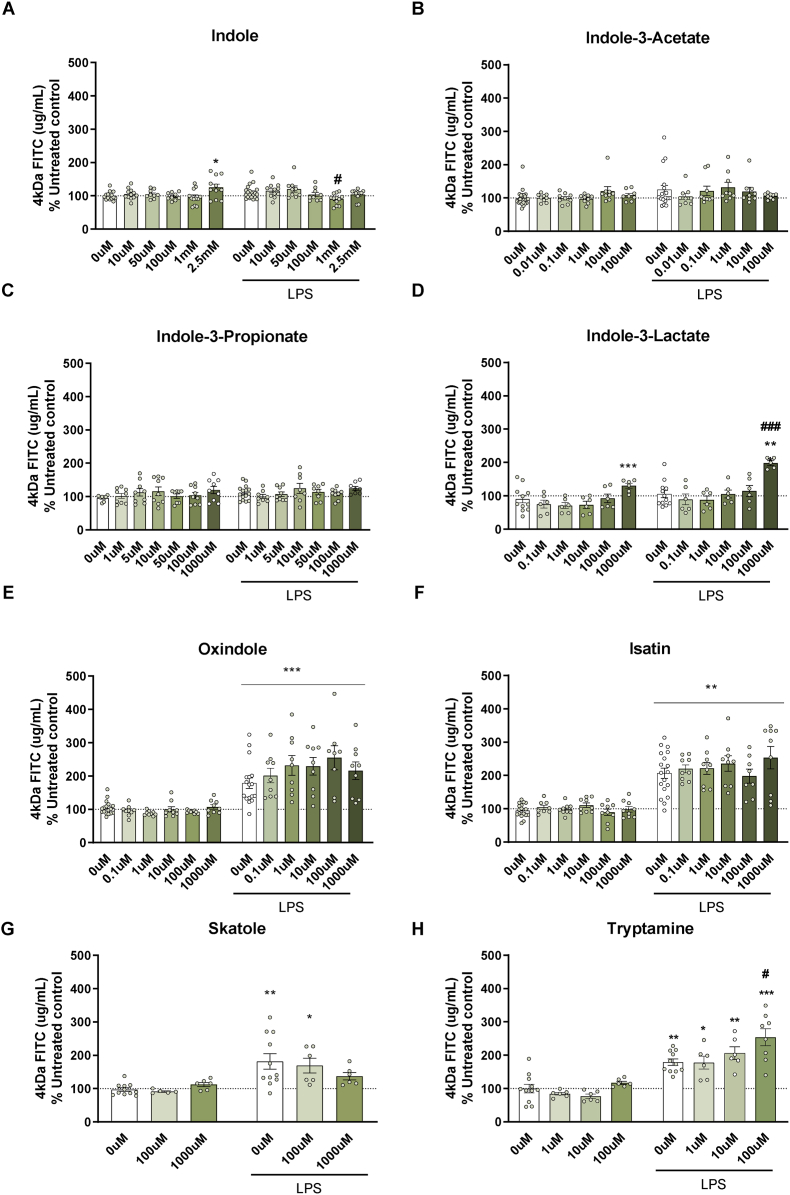
**FITC 4 kDa Permeability of T84 cells following exposure to tryptophan metabolites with and without a 24 h exposure of LPS.** (A) Indole, (B) indole-3-acetate, (C) indole-3-propionate, (D) indole-3-lactate, (E) oxindole, (F) isatin, (G) skatole, (H) tryptamine. Data are mean ± SEM; two-way ANOVA followed by Dunnet's post hoc compared to untreated groups. ∗p < 0.05, ∗∗p < 0.01, ∗∗∗p < 0.001 metabolite compared to 0 μM control, #p < 0.05, ##p < 0.01, ###p < 0.001 compared to 0 μM LPS.

Regarding FITC permeability of T84 cells (Fig. 4), LPS insult increased FITC permeability in indole-3-acetate, indole-3-lactate, oxindole, isatin, skatole, and tryptamine [LPS, F (1,122) = 7.713, p = 0.006 in indole-3-acetate; LPS, F (1,70) = 13.257, p < 0.001 in indole-3-lactate; LPS, F (1,111) = 124.924, p < 0.001 in oxindole; LPS, F (1,113) = 85.218, p < 0.001 in isatin; LPS, F (1,39) = 24.061, p < 0.001 in skatole; LPS, F (1,52) = 97.295, p < 0.001 in tryptamine]. Furthermore, a significant effect of the metabolite treatment was also observed with indole, indole-3-lactate, and tryptamine [indole, F (5,129) = 3.654, p = 0.003; indole-3-lactate, F (5,70) = 10.069, p < 0.001; tryptamine, F (3,52) = 7.079, p < 0.001]. No significant changes were seen after treatment with the other metabolites. Of note, there was an interaction between indole and LPS (F (5,129) = 2.61, p = 0.027). Post-hoc analysis showed a protective effect of indole at 1 mM compared to LPS-treated cells, whereas indole (2.5 mM), indole-3-lactate (1000 μM), and tryptamine (100 μM) increased the permeability of the gut barrier. Of interest, the concentrations used did not alter cell viability of T84 cells (Supplementary Fig. 3).

## 4 Discussion

In this current study, we revealed that specific microbial indole metabolites distinctly modulate the integrity of both intestinal and brain barriers in a dose- and compound-dependent manner. These metabolites, previously shown to be regulated by acute stress (Gheorghe et al., 2024), offer important insights into the key stress-regulated tryptophan metabolites that can influence host brain and gut barriers.

Tryptophan is an essential amino acid and precursor to serotonin the levels of which have been also shown altered by acute stress (Lyte et al., 2020). It plays a key role in this barriers interplay, being metabolized into a variety of bioactive compounds that impact both gut and brain physiology (Gheorghe et al., 2019). Using established *in vitro* models, we demonstrated that indole-derived tryptophan metabolites regulate both transcellular and paracellular permeability of gut and brain barriers in a dose- and compound-dependent manner. For instance, indole, indole-3-acetate, and skatole prevented LPS-induced barrier disruption in T84 cells. These results are consistent with prior findings showing indole-mediated increases in TEER in Caco-2 cells (Armand et al., 2022), and with evidence supporting the role of indole-3-acetate in maintaining gut barrier integrity and reducing LPS-stimulated inflammation in macrophages (Krishnan et al., 2018). In addition, indole producing bacteria enhance epithelial barrier function in germ-free mice, supporting our findings (Shimada et al., 2013). In contrast to what has been shown by other studies (Modoux et al., 2021), indole-3-propionate and tryptamine appeared to exacerbate LPS-induced disruption in a dose and assay-dependent manner. Tryptamine, for instance, has been associated with both anti-inflammatory effects and metabolic dysregulation, including reduced short-chain fatty acids (SCFA)-producing microbes and impaired insulin sensitivity (Bhattarai et al., 2020; Otaru et al., 2024; Zhai et al., 2023). These differences might be due to cell type, dose-range of concentrations and type of insult. Overall, these microbial metabolites can influence several aspects of gut physiology, the dysregulation of which has been implicated in a range of disorders (Turner, 2009), emphasizing the relevance as potential drug targets.

In contrast to better-characterized roles in gut barrier, the evaluation of the effects of microbial metabolites on the BBB remain largely understudied, suggesting a gap in our current knowledge regarding their role at the BBB (Knox et al., 2022b). To our knowledge, this is the first study to demonstrate that specific doses of indole, indole-3-propionate and tryptamine can enhance brain endothelial integrity, often in contrast to their activity at gut barrier cell model, highlighting location-specific action of these metabolites. Aligning with this data, indole-3-propionate, a metabolite that can cross the BBB (Pappolla et al., 2021), has been shown to reduce brain injury in neonatal rats (Zhao et al., 2022), but further studies are needed to elucidate precise mechanism of regulation of the BBB.

Notably, the mechanism of action of these metabolites could be through the activation of the aryl hydrocarbon receptor (AhR) (Ma et al., 2020), a transcription factor involved in immune modulation and maintenance of barrier integrity in both the gut and the brain (Gheorghe et al., 2019; Vázquez-Gómez et al., 2023). For example, tryptamine has been shown to suppress LPS-induced inflammation in macrophages via AhR (Agista et al., 2022). Our findings suggest a more nuanced role depending on cell type, concentration, and context. Importantly, the intestinal and brain barriers differ in their structure and immunological responsiveness. While the intestinal epithelium serves as an immunomodulatory, selectively permeable gateway, the BBB is formed by tightly connected endothelial cells with limited pro-inflammatory cytokine production (Spadoni et al., 2017), which may underlie the differential responses observed in our models.

The mechanisms regulating paracellular and transcellular transport differ; for example, in cytokine-based studies, specific cytokines significantly altered TEER, whereas FITC-dextran permeability remained unchanged; a discrepancy attributed to the differential involvement of specific tight junction components such as ZO-1 or claudins, which predominantly modulate paracellular permeability (Prasad et al., 2005; Greene et al., 2019; Weber et al., 2010). Therefore, while both TEER and FITC-dextran assays determine complementary aspects of barrier function, TEER reflects overall tight junction integrity and ion flow across the monolayer, whereas FITC-dextran represents the permeability through the paracellular route. This distinction may explain some of the assay- and compound-dependent differences observed in our study.

Reported physiological concentrations in healthy adults range from 0.481 μM (0.291–1.095 μM) to 2.85 ± 1.71 μM (HMDB: https://hmdb.ca/), although quantitative data on blood concentrations of many tryptophan metabolites remain limited. Supplementation with oral tryptophan can increase indoles to ∼ 5 μM and in kidney disease patients, plasma concentrations of indole-3-acetic acid have been detected up to 13.50±13.8 μM in adults (Paats et al., 2020; Rueda et al., 2024). While some of the concentrations used in bEnd.3 cells in our study exceed physiological levels, they are consistent with previous studies (Zhao et al., 2022; Huang et al., 2022; Serger et al., 2022). Interestingly, the most pronounced effects were observed at 0.1, 1, and 10 μM levels that align closely with reported physiological ranges.

Although *in vitro* models of stress differ significantly from pre-clinical or clinical models, one proposed mechanism for barrier disruption under stress involves pro-inflammatory signaling, particularly through the activation of NF-κB via TLR-4 (Welcome and Mastorakis, 2020). Since LPS activates this pathway, its use as a barrier disruptor compromising both transcellular and paracellular integrity provides a relevant model for studying microbial metabolite interactions with both BBB and gut barrier integrity under stress (Welcome and Mastorakis, 2020; Stephens and von der Weid, 2020).

While we demonstrated barrier-modulatory effects of these metabolites, these monolayers used cannot fully recapitulate the complexity of host-microbiome interactions or cellular diversity. Future studies using *in vivo*, or 3D organoids derived from stem cells (Haddad et al., 2023; Lee et al., 2022; Rosell-Cardona et al., 2025b) should address the mechanisms underlying microbial metabolites effects on barriers integrity, particularly in stress-related conditions.

In conclusion, this study presents a systematic screening of microbial tryptophan metabolites, selected based on their modulation in response to acute stress, across a range of concentrations in two key barrier cell models. The findings identify a subset of bioactive metabolites and concentration ranges that consistently influence barrier integrity. This resource will be valuable for future research, helping to prioritize the most promising candidates for in-depth functional and mechanistic studies. Given the dynamic nature of the circulating microbial metabolite profile in humans, this approach is crucial for capturing the complexity of microbial signaling within the microbiota-gut-brain axis. These findings reinforce the idea that microbial metabolites are not universally beneficial or detrimental but instead exert context-dependent effects that in the future could be harnessed for precision microbiome-targeted therapies.

## Author contributions

Conceptualization, C.R.C., S.J.L., E.K., J.F.C., and G.C; methodology C.R.C., P.S., S.J.L., E.K., and E.T.; Investigation, C.R.C., P.S., S.J.L., E.K., E.T.; writing-original draft, C.R.C., E.K.,; writing-review & editing, C.R.C., S.J.L., E.K., E.T., M.S.G., N.K.L., M.R.A., S.K.S, J.F.C., G.C.; funding acquisition, M.S.G., N.K.L., J.F.C., G.C.

## Declaration of interests

The research was conducted in the APC Microbiome Ireland which is funded by Science Foundation Ireland (now Research Ireland, SFI/12/RC/2273_P2). This project in part is a collaborative agreement (FA9550-17-1-0016) funded by European Office of Aerospace Research and Development, Air Force Office of Scientific Research and 711 Human Performance Wing, Air Force Research Laboratory.

The views expressed are those of the authors and do not reflect the official views of the United States Air Force, nor the Department of Defense. Mention of trade names, commercial products, or organizations do not imply endorsement by the U.S. Government (Distribution Statement A. Approved for public release. Distribution is unlimited. Case Number: AFRL-2024-6130, Nov 20, 2024).

Cristina Rosell-Cardona has received funding from the European Union's Horizon 2020 Research and Innovation Programme under the INSPIRE COFUND Marie Skłodowska-Curie grant agreement No. 101034270.

Sarah-Jane Leigh reports financial support was provided by Irish Research Council Postdoctoral Fellowship (GOPID/2021/298). Maria R. Aburto reports financial support was provided by European Research Council (ERC_StG_RADIOGUT (project 101040951)) and by Science Foundation Ireland Public Fellowship Programme (SFI‐IRC Pathway Program SFI21/PATH‐S/9424). The funding sources did not influence or constrain the study design, the collection, analysis, and interpretation of data, or the writing of the manuscript.

John F. Cryan reports a relationship with Bromotech that includes: speaking and lecture fees; and with Reckitt, Nutricia Research BV; DuPont Nutrition & Bioscience that includes: research funding grants. John F. Cryan reporsts a relatiponship with Nestle that includes: speaking and lecture fees and research funding grants. Gerard Clarke reports a relationship with Janssen Pharmaceuticals Inc that includes: speaking and lecture fees. Gerard Clarke reports a relationship with Probi AB that includes: speaking and lecture fees. Gerard Clarke reports a relationship with Boehringer Igelheim GmbH that includes: speaking and lecture fees. Gerard Clarke reports a relationship with Apsen Farmaceutica SA that includes: speaking and lecture fees. Gerard Clarke reports a relationship with Pharmavite LLC; Fonterra Research and Development; Tate and Lyle and Nestle that includes: research funding grants. Gerard Clarke reports a relationship with Yakult Central Research Institute for Microbiological Research that includes: consultancy. Gerard Clarke reports a relationship with Heel Pharmaceuticals that includes: consultancy, speaking and lecture fees. Gerard Clarke reports a relationship with Zentiva group that includes: consultancy. Sarah Kittel-Schneider has received speaker's honoraria from Medice Arzneimittel Pütter GmbH, Takeda and Janssen. This support neither influenced nor constrained the contents of this manuscript. CRC has nothing to disclose. This support neither influenced nor constrained the contents of this manuscript. All other authors declare no competing interests or personal relationships that could have appeared to influence the work reported in this paper.
