# Supplementary material for: Microbial tryptophan metabolites modulate blood-brain and gut barriers in vitro
Source: Neurosci Appl. 2025 Nov 21;5:106876. doi: 10.1016/j.nsa.2025.106876 (PMC13145374; doi:10.1016/j.nsa.2025.106876)
Supplement: Multimedia component 1 [file mmc1.docx]

**SUPPLEMENTARY FIGURE LEGENDS**

***Supplementary Figure 1. Optimisation of LPS in bEnd.3 cells.*** (A) *Transendothelial electrical resistance of* *bEnd.3 following LPS exposure, (B) FITC 4kDa Permeability of bEnd.3 cells following exposure of LPS, (C) Cell viability of bEnd.3 cells following exposure of LPS.*

***Supplementary Figure 2. Cell viability of bEnd.3 cells following exposure to tryptophan metabolites with and without a 24 h exposure of LPS.*** *(A) Indole, (B) indole-3-acetate, (C) indole-3-propionate, (D) indole-3-lactate, (E) oxindole, (F) isatin, (G) skatole, (H) tryptamine. Data are mean ± SEM two-way ANOVA followed by Dunnet’s post hoc compared to untreated groups. *p<0.05, **p<0.01, ***p<0.001 metabolite compared to 0 μM control.*

***Supplementary Figure 3. Cell viability of T84 cells following exposure to tryptophan metabolites.*** *(A) Indole, (B) indole-3-acetate, (C) indole-3-propionate, (D) indole-3-lactate, (E) oxindole, (F) isatin, (G) skatole, (H) tryptamine. Data are mean ± SEM two-way ANOVA followed by Dunnet’s post hoc compared to untreated groups. *p<0.05, **p<0.01, ***p<0.001 metabolite compared to 0 μM control.*

**SUPPLEMENTARY FIGURE 1**

**SUPPLEMENTARY FIGURE 2**

**SUPPLEMENTARY FIGURE 3**
